# The Additional Secondary Phase Correction System for AIS Signals

**DOI:** 10.3390/s17040736

**Published:** 2017-03-31

**Authors:** Xiaoye Wang, Shufang Zhang, Xiaowen Sun

**Affiliations:** Information Science and Technology College, Dalian Maritime University, Dalian 116026, China; sfzhang@dlmu.edu.cn (S.Z.); sunxiaowen_1984@163.com (X.S.)

**Keywords:** automatic identification system (AIS) signal, additional secondary phase factor (ASF), real-time correction, correction system

## Abstract

This paper looks at the development and implementation of the additional secondary phase factor (ASF) real-time correction system for the Automatic Identification System (AIS) signal. A large number of test data were collected using the developed ASF correction system and the propagation characteristics of the AIS signal that transmits at sea and the ASF real-time correction algorithm of the AIS signal were analyzed and verified. Accounting for the different hardware of the receivers in the land-based positioning system and the variation of the actual environmental factors, the ASF correction system corrects original measurements of positioning receivers in real time and provides corrected positioning accuracy within 10 m.

## 1. Introduction

At present, maritime radio navigation primarily relies on the Global Navigation Satellite System (GNSS); however, relying solely on GNSS has identified a potential safety hazard [1,2,3,4]. To guarantee coastal navigation safety, the International Maritime Organization (IMO) has explicitly stated that all ships should mandatorily install positioning and navigation systems of space-based and land-based backups [5,6,7]. The Automatic Identification System (AIS) is a shipborne navigation system mandatorily installed by the IMO. To realize the mutual recognition and information interaction between individual vessels and vessels to base stations, AIS—in the very high frequency (VHF) band—could send the positioning information of vessels, as well as the static and dynamic identification information of other vessels, to surrounding vessels and base stations with Self-Organized Time Division Multiple Access (SOTDMA) [8,9,10]. Presently, major coastal states and regions in the world have established comparatively perfect AIS shore-based systems to obtain vessel information [11]. As the positioning information of the shipborne AIS equipment originates from the GNSS positioning module embedded in the equipment [12,13], once the GNSS fails, the AIS will not operate normally, which presents a serious threat to navigational safety. Under the IMO’s e-navigation strategy [14,15], member countries would be called on to use AIS R-mode land-based positioning technology, which uses AIS base stations as positioning reference points [16,17]. Research in this area has been conducted since 2012 by the Navigation Institute of Dalian Maritime University in Dalian, China, where the authors are based. Using the existing functions of AIS to increase the autonomous positioning function, AIS with an autonomous range positioning function not only meets the functional requirements of the land-based navigation system, but there are also savings on the large establishment costs and maintenance system, and it would be unnecessary to load new shipborne equipment. Therefore, this study has a wide range of applications.

In the radio positioning system for the range positioning method, the distance measurement is obtained mainly by measuring the signal transmission delay [18,19,20,21]. The existing AIS is a pure communication system and its purpose is to receive source information without distortion at the receiving end, not to consider the measurement requirements of the signal transmission delay. To increase the positioning function in AIS, it is necessary to find and identify the channel information carried by the AIS communication signal under the condition of the existing AIS communication source information. According to the signal measurement technique mentioned in Reference [22], the positioning accuracy of the verification system for the AIS Autonomous Positioning System (AAPS) was more than 100 m (2σ) with a geometric dilution of precision (GDOP) of less than 1.5 and it could not meet the requirements of positioning accuracy. In land-based positioning systems, radio waves are influenced by the transmission medium and it limits the positioning accuracy of the system to a large extent [23,24], which makes it necessary to carry out studies on the additional secondary phase factor (ASF) correction. The radio transmission delay between two points could be expressed as the primary phase factor (PF) delay, the secondary phase factor (SF) delay and the ASF delay [25]. The PF delay is the sum of the net propagation time from the transmitting antenna to the receiving antenna when the signal is transmitting in the atmosphere [26]. The SF delay is the additional delay generated by the signal transmitting on the sea surface [26]. The ASF delay is the final delay generated by the signal transmitting in a transmission medium with a different conductivity [26]. At present, for the AIS signal, there are no available theoretical models to directly predict the delays of the SF and the ASF, and we could not accurately distinguish between the two in the actual experiments and application systems. However, the test system presented in this paper could measure the sum of the SF delay and the ASF delay in real time, which could be used in the practical application. By considering the sea surface as one of the transmission mediums, the SF and the ASF are collectively referred to as the ASF in the context of this paper, and it is the sum of the additional phase delay caused by the transmission medium that is different from the atmosphere. The definition used in this paper not only has general theoretical significance, but is also of practical value. The current research in ASF correction of land-based positioning systems commonly uses the Loran-C system [27,28,29,30,31]; however, the transmission mode and frequency of AIS are not the same as those of the Loran-C system, and thus the ASF correction technology used in Loran-C is not completely applicable to AIS. Therefore, this paper aims to design an ASF correction system based on the autonomous positioning feature of AIS. To remove the influence of the ASF, the main research of this paper includes the theoretical and experimental research and the actual positioning error correction experiments using the developed system. For the theoretical component, the implementation principle and development method of the ASF correction system are provided in detail. In the experimental research, a devised and developed ASF correction system—composed of one transmitter and two receivers—was erected to carry out real-time tests in a fixed coastal area and collect large amounts of test data. By using the measured data to analyze the propagation characteristics of the maritime VHF signal, it could be verified that study on the ASF correction algorithm of maritime VHF signals and the hardware platform could be provided for the real-time publication of the ASF correction parameters. Finally, the actual positioning error correction experiments were carried out in the actual sea surface environment. The system was able to measure the transmission delay between two known points in real time and compare it with the known vacuum transmission delay between the two points to obtain the correction parameters of the real-time transmission delay. The correction parameters could then be broadcast to users within the effective range to improve the positioning accuracy of users so that the corrected positioning accuracy could meet 10 m (95%). 

In future research, based on the correction range of the ASF, several developed ASF correction systems could be erected in a coastal area to realize the full coverage of the coastal area with ASF correction technology. In the covered sea area, the AIS autonomous positioning users could correct the ASF to meet the required positioning accuracy. Based on the construction of the ASF correction systems, the user data could be corrected in real time, while the ASF data could be saved and counted for a long time to constantly improve the correction models. Once the measurement signal fails, the current correction parameter of the ASF can be obtained through the correction models before being broadcast to users to achieve the double-backup ASF correction parameters to ensure the integrity of the users’ positioning accuracy and ensure navigational safety.

This paper on the ASF correction system is organized as follows: Section 2 discusses the overall design of the ASF correction system, including the basic structure and measuring principle of the correction system, and provides the selection method and signal format of the VHF signal for testing. The development process of the transmitter and receiver for the ASF correction system is given in Section 3, and will introduce the signal generating module of the transmitter and the signal processing module of the receiver. Section 4 provides the ASF data measured by the ASF correction system. The application of the ASF correction system in AIS is discussed in Section 5 and it analyzes the positioning error measured in the actual experiments. Finally, some conclusions are put forth in Section 6. 

## 2. The Overall Design of the Secondary Phase Correction System

### 2.1. The Basic Structure and Measuring Principle of the ASF Correction System

**Figure 1:**
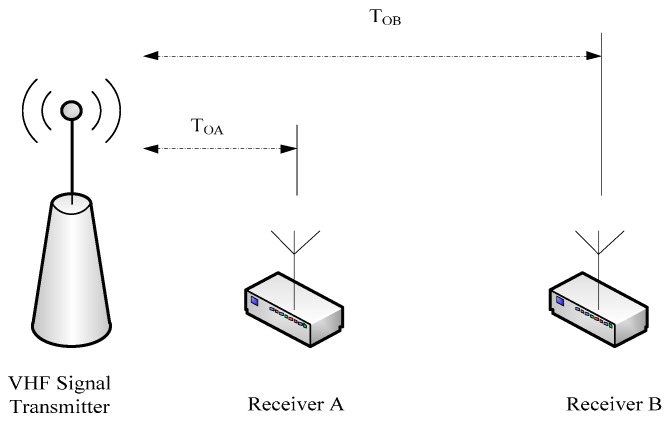
The basic structure of the additional secondary phase factor correction system.

The basic structure of the ASF correction system is shown in Figure 1. The system was applied in an area where a transmitter sent a VHF signal and two receivers received and processed the signal simultaneously to remove the effects of the hardware circuit [32]. To accurately measure the signal transmission delay, the synchronization between the transmit system and receiving systems needed to be kept by the atomic clock [33]. The ASF correction system needs to synchronize time between the transmitter and two receivers when the system is operating, and then the three subsystems (one transmit system and two receiving systems) work under the synchronous pulse per second (PPS) pulse control. The transmission delay of the test signal was obtained using the synchronous PPS pulse as a benchmark [34].

Receiver A was located very close to the transmitter (less than 10 m), and the moving range of Receiver B was the test area. *T_OA_* is the measured time delay of Receiver A, *T_OB_* is the measured time delay of Receiver B and they are expressed as per Reference [32].

(1) $$T_{OA}=PF_{OA}+SF_{OA}+ASF_{OA}+t_{rA}$$

(2) $$T_{OB}=PF_{OB}+SF_{OB}+ASF_{OB}+t_{rB}$$

where $PF_{OA}=\frac{d_{OA}}{c}$ is the PF delay of Receiver A; *d_OA_* is the distance from Receiver A to the transmitter; *c* is the speed of light; *SF_OA_* is the SF delay of Receiver A; *ASF_OA_* is the ASF delay of Receiver A; $t_{rA}$ is the equipment fixed delay of Receiver A and it can be eliminated by repeated measurements; $PF_{OB}=\frac{d_{OB}}{c}$ is the PF delay of Receiver B; *d_OB_* is the distance from Receiver B to the transmitter; *SF_OB_* is the SF delay of Receiver B; *ASF_OB_* is the ASF delay of Receiver B; and $t_{rB}$ is the equipment fixed delay of Receiver B, which is caused by the device itself and it can be eliminated by repeated measurements. Receiver A was very close to the transmitter so *SF_OA_* + *ASF_OA_* can be ignored. Therefore, the following formula can be given by Equations (1) and (2):

(3) $$T_{OB}-T_{OA}=(PF_{OB}-PF_{OA})+(SF_{OB}+ASF_{OB})+(t_{rB}-t_{rA})$$

So

(4) $$ASF_{OB}+SF_{OB}=(T_{OB}-T_{OA})-\frac{d_{OB}-d_{OA}}{c}-(t_{rB}-t_{rA})$$

In the formula, $T_{OB}-T_{OA}$ can be obtained by the actual measurement, and $\left(t_{rB}-t_{rA}\right)$ can be eliminated by repeated measurements. The positions of the transmitter and Receiver A are known, while the position of Receiver B can be acquired accurately by the real-time kinematic (RTK) test. After obtaining the $ASF_{OB}+SF_{OB}$ value in the test area, the ASF correction model was obtained using the combined ratio of the distance between the transmitter and receivers to the radio wave velocity, along with the variation of environmental factors.

### 2.2. The Selection Method and Signal Format of the Test Signal for the ASF Correction System

To precisely measure the transmission delay of the signal between the transmitter and receivers and obtain the propagation characteristics of the AIS signal in the actual environment using the statistics, the test signal is required to meet the following conditions:

1. The test signal should be as similar to the actual AIS signal as possible, so the propagation characteristics of the actual AIS signal can be approximately obtained by the propagation characteristics of the test signal;
2. Unlike the AIS system using time division multiple address (TDMA) mode, the transmitter and receivers of the ASF correction system should keep continuously working to provide a more accurate measurement of the time delay. Therefore, the ASF correction system at work should ensure that it does not affect the normal operation of the actual AIS system. This requires the central frequency point of the test signal to be different from the central point of the actual AIS signal and the transmission power should be as low as possible.
3. The format of the test signal should be appropriate for measuring the transmission delay, and the modulation and demodulation of the signal should be easy to realize as far as possible.

To meet the above conditions, the characteristics of the selected test signal are as follows.

1. The test signal modulates the carrier signal by way of binary phase shift keying (BPSK) using the pseudorandom sequence. This type of signal was helpful for accurately measuring the transmission delay of the signal and for the current GNSS systems to measure the transmission delay. Thus, the modulation and demodulation of BPSK were very easy to obtain.
2. The bit rate of the pseudorandom sequence was set to 10,200 bps, close to the bit rate of the actual AIS signal, which is 9600 bps. The frequency of the carrier signal was set to 160 MHz and the transmit power was set to 100 mW. This type of signal was similar to the actual AIS signal, but did not affect the normal work of the AIS system.
3. A pseudorandom sequence length of 255 chips is close to an AIS signal message length of 256 bits and the primitive polynomial of the pseudorandom sequence is $x^{8}+x^{4}+x^{3}+x^{2}+1$. Furthermore, the bit rate of the pseudorandom sequence was 10,200 bps, and it can guarantee 40 complete cycles in one second.

This design was suitable for the accurate measurement of the transmission delay of the signal by using the synchronized 1 pps pulse of the transmit-receive system as the time identification. The parallel detection method was used in the signal searching and tracking stage of the receivers. The first correlator produces the chip of a zero-phase delay, and other correlators increase half a chip phase delay in sequence, so that the searched phase delay will not exceed half a chip phase delay and improve the search efficiency. *K* correlators were selected in this paper (where *K* is odd), and the searched maximum value of the correlator was assigned to the $\frac{K+1}{2}$th correlator. The first to $\frac{K-1}{2}$th correlators in turn lead $\frac{K+1}{2}$th codes by the $\frac{K+1}{2}$th correlator and the $\frac{K+3}{2}$th to *K*th in turn lags $\frac{T_{b}}{K-1}$ codes by the $\frac{K+1}{2}$th correlator. It makes full use of the existing correlators that use the above method and can simplify the complexity of the circuit.

The capture signal of the correlator is expressed as [35]

(5) $$s_{r}(t)=A_{r}b(t-t_{r})\cos(\omega_{c}t+\theta_{r})$$

where $A_{r}$,$t_{r}$,$\theta_{r}$ are the amplitude, the time delay and the phase of the capture signal, respectively; $\omega_{c}$ is the angular frequency of the intermediate frequency (IF) signal, which is equal to the angular frequency of the local carrier; b(t) is the pseudorandom sequence $b(t)=\sum_{n=1}^{N}b_{n}g(t-nT_{b})$; *N* is the length of the pseudorandom sequence; $T_{b}$ is the symbol interval; $b_{n}$ is the differential non return to zero(DNRZ) code generated by the pseudorandom sequence. The local carrier signal is $c_{0}(t)=\cos(\omega_{c}t+\theta_{0})$, $\theta_{0}$ is the phase of the local carrier. Assume that the M sequence signal is reproduced by the receiver in the capture time where $s_{0}(t)={\overset{\wedge }{A}}_{0}b(t-\overset{\wedge }{\tau_{0}})$ and ${\overset{\wedge }{A}}_{0}$, $\overset{\wedge }{\tau_{0}}$ are the amplitude and the time delay of the local reproductive signal of the receiver, respectively. The correlation calculation is performed by the local reproductive M sequence signal and the product that is the received signal and the local carrier signal [36], and the obtained correlation function is

(6) $$R_{I}(\Delta \tau )=\frac{1}{2}\cos(\theta_{r}-\overset{\wedge }{\theta_{0}})R(\Delta \tau )$$

(7) $$R(\Delta \tau )\left\{ \begin{array}{l} \frac{A_{r}}{\overset{\wedge }{A_{0}}}(1-\frac{\Delta \tau }{T_{b}}),\left|\Delta \tau \right|\leq T_{b}\\ 0,\left|\Delta \tau \right|>T_{b} \end{array}\right.$$

where Δτ is the delay corresponding to the signal reproductive moment, $\Delta \tau =\tau_{r}-\overset{\wedge }{\tau_{0}}$. To eliminate the influence of the phase in the correlation function, the output signal of the carrier signal generator can be divided into the in-phase branch and the orthogonal branch, and the sum of squares of the correlation value of the two branches can be calculated.

(8) $$\begin{array}{c} R_{I}(\Delta \tau )=\frac{1}{2}\cos(\theta_{r}-\overset{\wedge }{\theta_{0}})R(\Delta \tau )\\ R_{Q}(\Delta \tau )=\frac{1}{2}\sin(\theta_{r}-\overset{\wedge }{\theta_{0}})R(\Delta \tau ) \end{array}$$

(9) $$\begin{array}{cc} R_{s}^{2}(\Delta \tau ) & =R_{I}^{2}(\Delta \tau )+R_{Q}^{2}(\Delta \tau )\\ & ={\left[\frac{1}{2}\cos(\theta_{r}-\overset{\wedge }{\theta_{0}})R(\Delta \tau )\right]}^{2}+{\left[\frac{1}{2}\sin(\theta_{r}-\overset{\wedge }{\theta_{0}})R(\Delta \tau )\right]}^{2}\\ & =\frac{1}{4}R^{2}(\Delta \tau ) \end{array}$$

(10) $$R_{s}(\Delta \tau )=\frac{1}{2}R(\Delta \tau )$$

If the maximum value of the signal captured by the correlator is $R_{M}(\Delta \tau_{M})$, then $\overset{\wedge }{\tau_{0}}$ is the delay of the local reproductive signal captured at the maximum value moment, where $\Delta \tau_{M}=\tau_{M}-\overset{\wedge }{\tau_{0}}\leq \frac{T_{b}}{2}$ and $\tau_{M}$ is the delay of the maximum value of the signal. $R_{M}(\Delta \tau_{M})$ can be separate lead and lag $\frac{1}{K-1}$ codes, and then there are $\frac{K-1}{2}$ correlation values in the $\frac{T_{b}}{2}$ time range. The output *e*(*t*) of the calculation processing module can be obtained by the lead correlation value minus the lag correlation value, and the tracking error is:

(11) $$e(t)=\left\{ \begin{array}{l} \frac{A_{r}}{\overset{\wedge }{A_{0}}T_{b}}\sum_{i=1}^{\frac{K-1}{2}}(\left|\Delta \tau_{M}-\frac{iT_{b}}{K-1}\right|-\left|\Delta \tau_{M}+\frac{iT_{b}}{K-1}\right|),-\frac{(K-1-2i)T_{b}}{2(K-1)}\leq \tau_{M}\leq \frac{(K-1-2i)T_{b}}{2(K-1)}\\ 0,others \end{array}\right.$$

**Figure 2:**
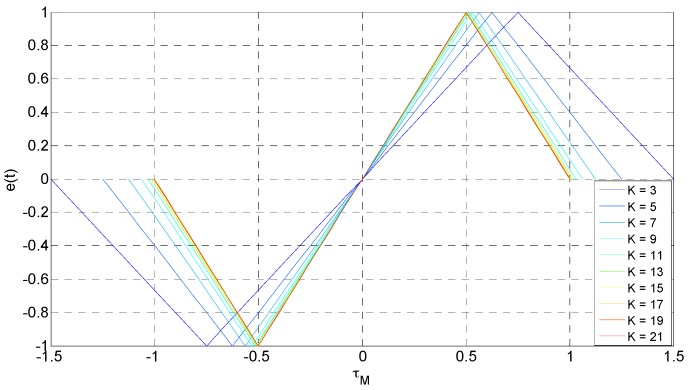
The relationship between the output of the computer processing module and tracking error.

Figure 2 shows the relationship between $\Delta \tau_{M}$ and the output of the calculation processing module which is obtained by selecting different *K* values.

## 3. The Design of the Transmitter and the Receiver

**Figure 3:**
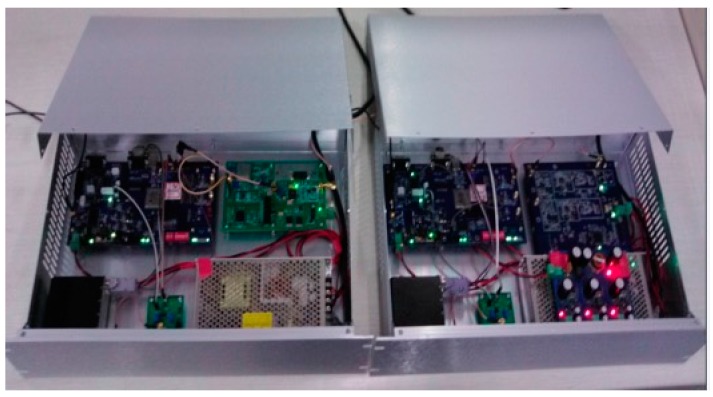
The internal configuration diagram of the transmitter and the receiver for the ASF correction system.

The transmitter and the receiver of the developed ASF correction system look identical and have the dimensions of 111 mm (h) × 482.6 mm (l) × 300 mm (w), as shown in Figure 3.

**Figure 4:**
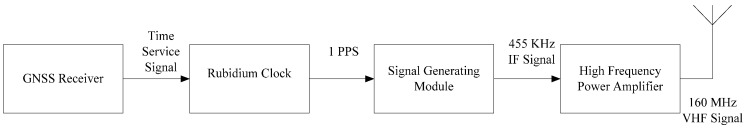
The structure diagram of the transmitter in the ASF correction system.

The transmitter was primarily composed of a GNSS disciplined rubidium clock, the converter plate of a rubidium clock signal, and the signal generating module based on Field Programmable Gate Array (FPGA), a high-frequency power amplifier board (Figure 4).

The accurate synchronization between the transmitter and receivers was achieved by a GNSS disciplined rubidium clock. The converter plate of the rubidium clock signal converts the level of the rubidium clock to meet the output signal level required by the FPGA chip. The signal generating module (based on FPGA) transmits the BPSK modulated signal, which is convenient to position. The high-frequency power amplifier board amplifies the power of the modulated VHF signal to 12.5 W to meet the transmission power of an AIS transmitter required by the IMO.

**Figure 5:**
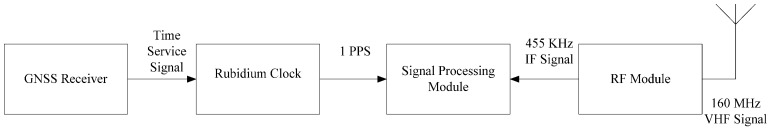
The structure diagram of the receiver in the ASF correction system.

The receiver was mainly composed of a GNSS disciplined rubidium clock, the converter plate of a rubidium clock signal, and the signal processing module was based on the FPGA and the radio frequency (RF) circuit (Figure 5). 

The accurate synchronization between the receivers and the transmitter was achieved by a GNSS disciplined rubidium clock of receivers. The functions of the converter plate of the rubidium clock signal of the receivers were the same as the transmitter. The signal processing module (based on FPGA) sampled analogue IF signals into digital signals, before the digital signal was input into the FPGA chip to complete the IF digital processing. The function of the radio-frequency circuit is to accomplish RF signal down-conversion into an IF signal.

This section will focus on the signal generating module of the transmitter and the baseband signal processing module of the receivers.

### 3.1. The Signal Generating Module of the Transmitter System

**Figure 6:**
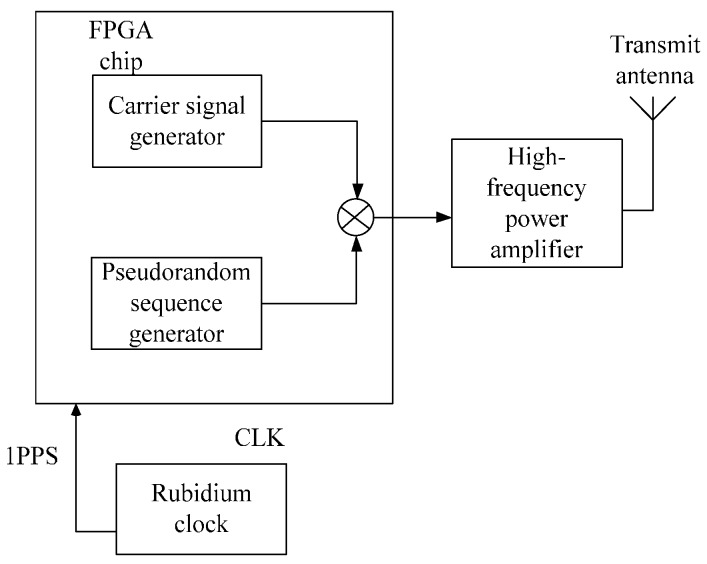
The structure of the baseband signal processing module based on FPGA.

The main function of the signal generating module based on the FPGA chip had three parts: a disciplining rubidium clock, a generating pseudorandom sequence and a modulating signal (Figure 6). As the integrity of the signal in the analog high-frequency circuit is difficult to obtain, and the high-speed processing capacity of FPGA chips becomes stronger, FPGA chips were used to accomplish the signal-modulated process. In the FPGA chip, a 160 MHz carrier signal is multiplied by 10,200 bps pseudorandom codes to obtain the BPSK-modulated output signal. The output signal was amplified by the high-frequency power amplifier and filtered, and then emitted by the antenna.

1. The disciplined rubidium clock module: The PPS signal was obtained by adjusting the divided frequency of the rubidium clock in this module to synchronize with the PPS signal provided by the external GNSS receiver. In the coarse adjustment stage, the disciplined rubidium clock module directly shifts the phase to the frequency division PPS signal. In the fine adjustment stage, this module adjusts the frequency of the rubidium clock by writing the frequency control word to the rubidium clock, to implement accurate synchronization between the phases of the two PPS signals.
2. The pseudorandom sequence generating module: The initial state of the eight-bit shift register all had a value of one, and the atomic clock provided a synchronized 1 PPS signal as a reset signal to take the register back to the initial state. The host clock signal of the system—provided by the atomic clock—was divided by obtaining the clock signal of 10,200 Hz that controlled the shift of the register. The length of the M sequence generated by this method was 255, and its primitive polynomial was $x^{8}+x^{4}+x^{3}+x^{2}+1$. The bit rate was 10,200 bps, and 40 cycles of the M sequence were generated per second.
3. The signal modulation module: The mode of the signal modulation was BPSK, and the carrier signal frequency was 160 MHz. Due to the high performance of the FPGA chip, the digital carrier used for the BPSK modulation of the signal was directly generated by the FPGA chip. In the modulating process, first, a pair of differential 160 MHz clocks was obtained by the frequency multiplication of the external 10 MHz signal. Next, as per the symbols of the pseudorandom sequence, the 160 MHz clock was output. If the code was “1”, a normal form was output. If the code was “0”, a differential form was output.

**Figure 7:**
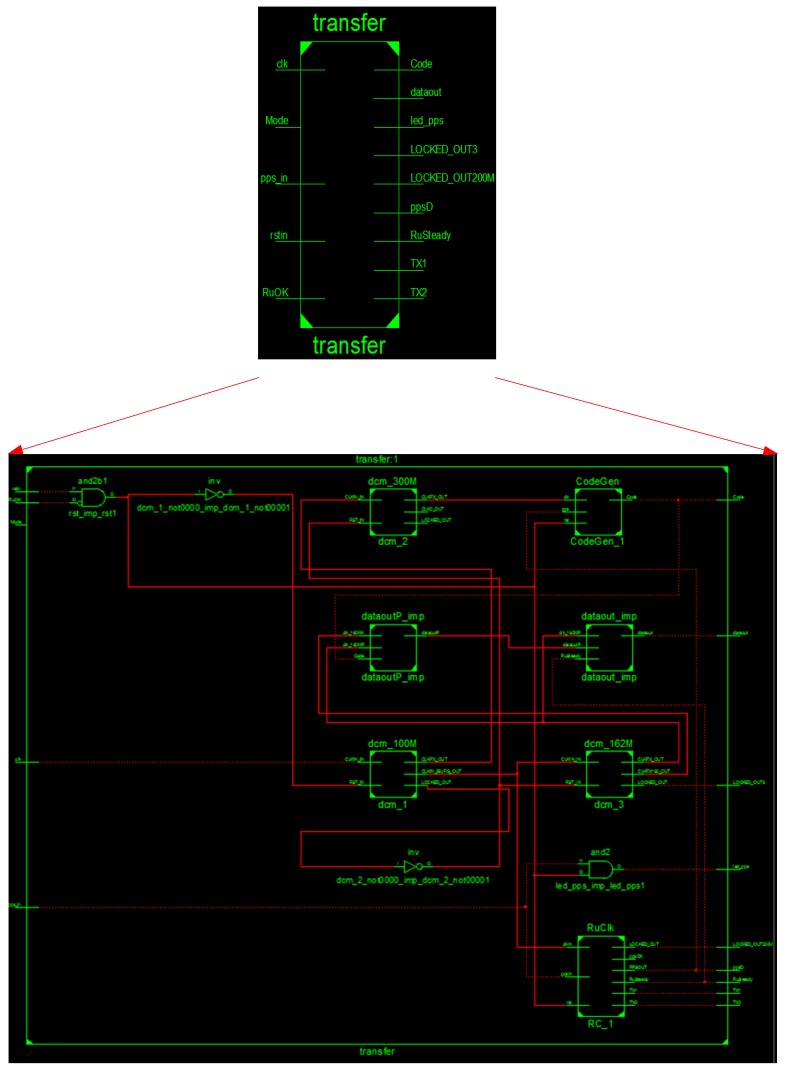
The schematic diagram of the signal generating module at RTL level based on FPGA.

In the realization of the above functions, the top-level and second-level schematic diagrams of the module at register transfer level (RTL) (based on the FPGA chip) are shown in Figure 7.

Using the theoretical simulation and practical test for the VHF signal, the transmitter in the ASF correction system could accurately and stably send the pseudorandom sequence when the modulation mode was BPSK, the carrier frequency was 160 MHz, the bit rate was 10,200 Hz and the sequence length was 255.

### 3.2. The Signal Processing Module of the Receiver System

**Figure 8:**
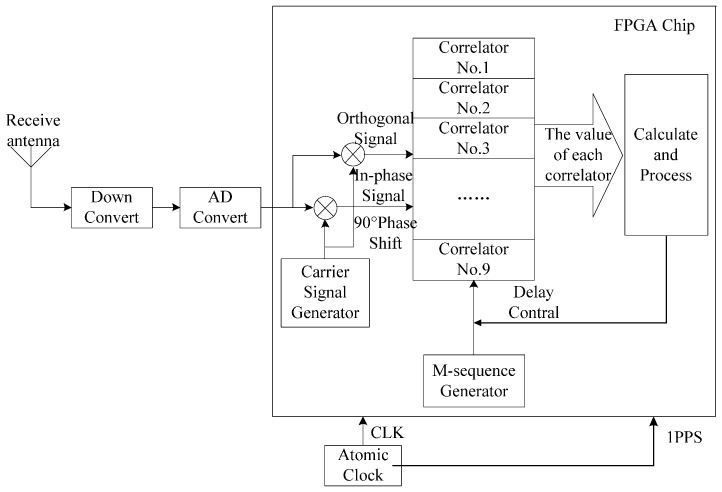
The structure of the baseband signal processing module based on FPGA.

The structure diagram of the baseband signal processing module of the receiver based on FPGA is shown in Figure 8. The signal received by the antenna was converted into a digital IF signal after down converting and A/D converting, and the signal was connected to the FPGA chip. The structure of the M sequence generator of the receiver system was identical to that of the transmitter system. The carrier signal generated by the carrier signal generator of the receiving system was the IF carrier, and the carrier signal was divided into in-phase and quadrature branches. It could ignore the influence of the carrier phase as the correlation values of the two branches were squared and summed. To achieve a balance between tracking accuracy and hardware simplification, this paper selected nine correlators to search and track the signal.

The receiver system was mainly composed of nine correlators. In the signal searching stage, the zero-phase delay code was generated by the first correlator and, in turn, the other correlators increased the phase delay of half a code, so that the nine correlators could search the signal where the phase delay was zero to four codes. As per the effective distance calculation of the actual transmission for the AIS signal, the time delay of zero to four codes covered all possible delay ranges of the signal, so the structure of the nine correlators chosen by the system could complete the signal searching. Additionally, in the signal tracking stage, the code phase of each correlator differed by one-eighth code in turn, and this structure of multi-correlators enhanced the signal-to-noise ratio (SNR) to improve the tracking sensitivity of the system. In the tracking stage, for the reproduced signal of each correlator and the received signal, there were code sequences of 40 cycles between two PPS pulses. The codes of one period were used for correlation calculations and accumulation to obtain the correlation value and the correlation value was used to adjust the carrier frequency. The codes of 38 periods were used for the correlation calculation and accumulation to obtain the correlation value used to adjust the code phase. The signal processing was carried out in the two redundant periods.

**Figure 9:**
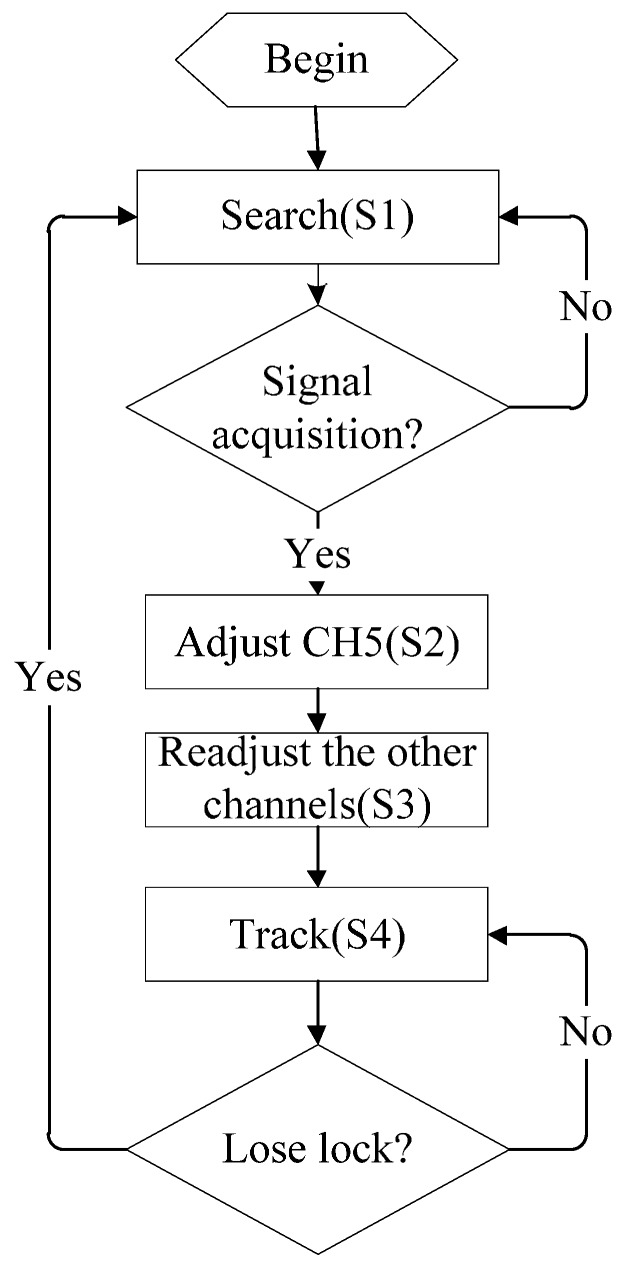
The work flow chart of the receive system.

The work flow chart of the receive system is shown in Figure 9, and the work process of the system includes four states: searching signal, adjusting the center channel, adjusting the other channels and tracking signal, represented as S1, S2, S3 and S4. They will be introduced separately in the following part.

S1: The searching signal state. In the searching state, it is necessary to carry out two-dimensional searches of the carrier frequency and code phase. The code phase interval of nine correlators was set at half a code, and the code phase of the first correlator was set at the synchronous code phase with zero delay. In the case of the accurately reproduced carrier frequency, there was a significant correlation peak in one of the nine correlators. The system then determined the signal captured and entered the state of S2, where the code phase delay of the correlator was the rough transmission delay of the signal.
S2: Adjusting the center channel. As per the correlation results of the nine correlators given by the state of S1, the maximum correlation value corresponding to the code phase of the correlator was set at the center channel, namely the code phase of CH5. The system enters the state of S3.
S3: Adjusting the other channels. In the case of the code phase of the center channel CH5 (determined by the state of S2), the code phases of the other channels were set. CH4, CH2, CH3, and CH1 (four channels) in turn lead one-eighth, a quarter, three-eighths and a half codes before the CH5 channel; CH6, CH7, CH8, and CH9 (four channels) in turn lag one-eighth, a quarter, three-eighths and a half codes behind the CH5 channel, before the system enters the state of S4.
S4: The tracking signal state. In the tracking state, the correlation values of the four leading channels, CH4, CH3, CH2, and CH1, and the correlation values of the four lagging channels, CH6, CH7, CH8, and CH9, were accumulated. The result obtained by subtracting the lagging accumulated value from the leading accumulated value was used to adjust the code phase of the CH5 channel. The use of the symmetry of the correlation characteristic curve of the pseudorandom code was used to achieve the real-time dynamic tracking of the pseudorandom code. Additionally, the correlation value of the CH5 channel was used to keep track of the carrier frequency. Finally, it determined whether to lose lock as per the magnitude of the correlation value given by the CH5 channel. If lost, the system will enter the state of S1 to search for the signal again.

**Figure 10:**
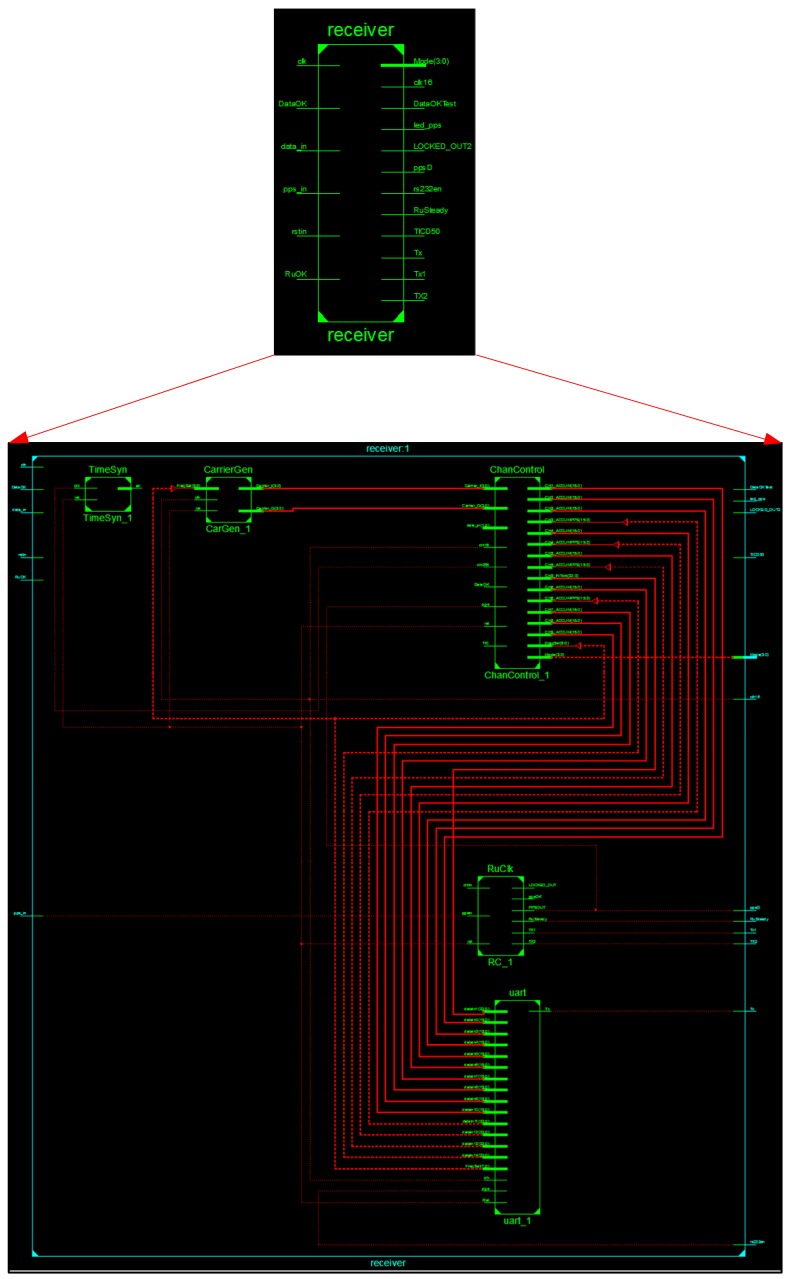
The schematic diagram of the baseband signal processing module at the RTL level.

In the realization of the above-mentioned functions, the top-level and second-level schematic diagrams of the baseband signal processing module at the RTL level based on the FPGA chip are shown in Figure 10.

The system will work under four different working conditions. Under all kinds of working conditions, as per the obtained correlation results, the system will dynamically adjust the carrier frequency and the code phase of nine correlators, and then the code phase of the nine correlators—including the time-delay information—is output by the serial communication module to achieve the time-delay value of the receive signal.

## 4. The Actual Test Results of the ASF Correction System

**Figure 11:**
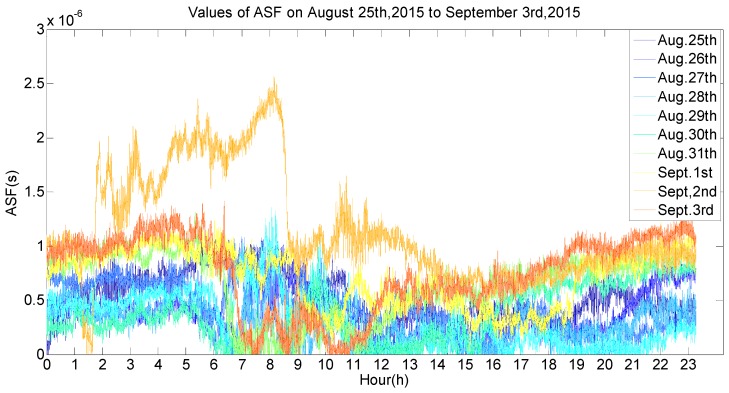
The ASF data from 25 August–3 September 2015.

To verify the functions of the ASF correction system, the system was tested in real time in the actual sea environment. The transmitter was installed at the Lingjing Hotel in Dalian, China (38°50′21.309′′ N, 121°30′46.005′′ E), and two receivers were separately installed in the Lingjing Hotel and the Fujiazhuang Ship Hotel in Dalian, China (38°51′52.660′′ N, 121°36′49.543′′ E). The transmitter continued to transmit a specific pseudorandom sequence, the receivers searched and tracked the received signal and adjusted counts. Next—ASF correction values were sent to the serial port in a value per second. The following data is from the period between 25 August–3 September 2015 (Figure 11), to discuss the ASF data obtained by the ASF correction system.

**Figure 12:**
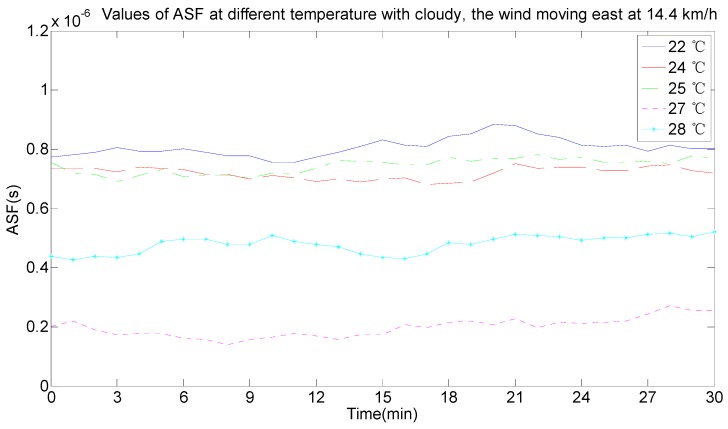
ASF values changing with different temperature conditions.

**Figure 13:**
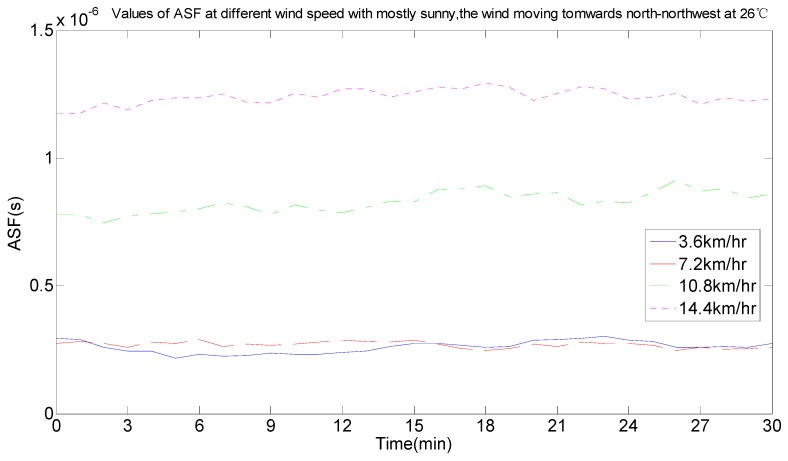
ASF values changing with different wind speed conditions.

**Figure 14:**
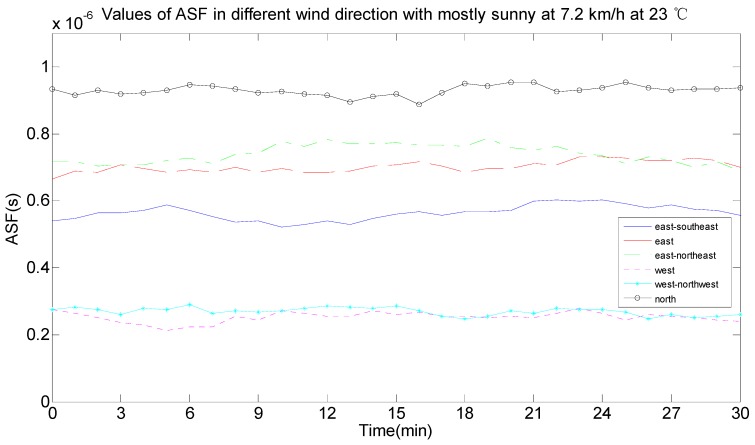
ASF values changing with different wind direction conditions.

**Figure 15:**
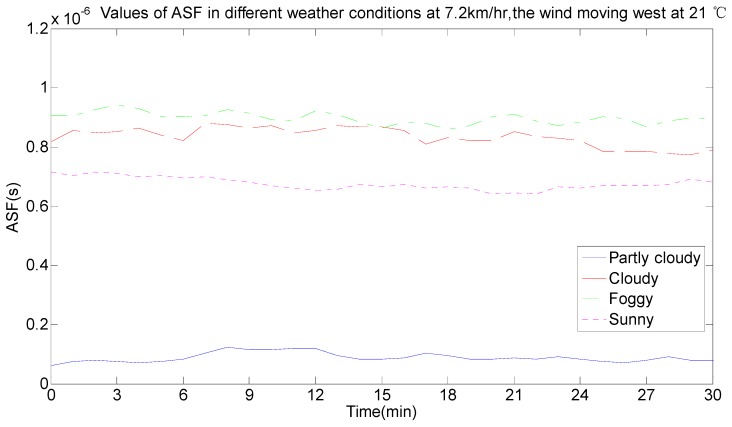
ASF values changing with different weather conditions.

As seen in Figure 11, the ASF data was not constant and changed every day. By comparing the real-time weather conditions, the ASF values were influenced by temperature, wind speed, wind direction and weather. The effect of the change conditions on the ASF values when the conditions of temperature, wind, wind direction and weather were the same were analyzed, as per the half-hour weather data classification and reference to the local real-time climate data. In Figure 12, the weather was cloudy, the wind speed was 14.4 km/h, the wind direction was east, and the ASF value changed with temperature changes at 22 °C, 24 °C, 25 °C, 27 °C, and 28 °C, and the data are summarized in Table 1. In Figure 13, the weather was partly cloudy, the wind direction was north-northwest, the temperature was 23 °C, and the ASF values changed with the wind speed, changing from 3.6 km/h, 7.2 km/h, 10.8 km/h, and 14.4 km/h, and the data is summarized in Table 2. In Figure 14, the weather was partly cloudy, the wind speed was 10.8 km/h, the temperature was 26 °C, and the ASF values changed with the wind direction, changing towards east-southeast, east, east-northeast, west, north-northwest, and north (Table 3). In Figure 15, the temperature was 21 °C, the wind direction was west, the wind speed was 7.2 km/h, and the ASF values changed with weather changes of partly cloudy, cloudy, foggy, and sunny (Table 4). 

From the information in Table 1—given the same circumstances of weather, wind speed and wind direction—the temperature affected the ASF values and its influence was as follows. The ASF values were larger and the ranges that needed correction were bigger when the temperature was low. According to the average value of the error correction in combination with the statistical probability of the error correction interval, the selected interval range makes the statistical probability as large as possible and the average value of the error correction was calculated in this interval range. The following results can be discussed:

(1) The ASF correction system provided an average correction of 229.0738 m for the original data at 22 °C.
(2) The ASF correction system provided an average correction of 243.6178 m for the original data at 24 °C.
(3) The ASF correction system provided an average correction of 276.525 m for the original data at 25 °C.
(4) The ASF correction system provided an average correction of 66.1956 m for the original data at 27 °C.
(5) The ASF correction system provided an average correction of 128.1657 m for the original data at 28 °C.

Therefore, a correction of 66.1968–276.525 m was provided by the developed ASF correction system under the current conditions.

From the information in Table 2—given the same circumstances of the weather, the wind direction and the temperature—the wind speed affected ASF values and its influence was that ASF values were larger and the ranges that needed correction were bigger when the wind speed was fast. According to the average value of the error correction in combination with the statistical probability of the error correction interval, the selected interval range makes the statistical probability as large as possible and the average value of the error correction was calculated in this interval range. The following results can be discussed:

(1) The ASF correction system provided an average correction of 41.0782 m for the original data with 3.6 km/h of wind speed.
(2) The ASF correction system provided an average correction of 54.8815 m for the original data with 7.2 km/h of wind speed.
(3) The ASF correction system provided an average correction of 284.662 m for the original data with 10.8 km/h of wind speed.
(4) The ASF correction system provided an average correction of 305.8199 m for the original data with 14.4 km/h of wind speed.

Therefore, a correction of 41.0782–305.8199 m was provided by the developed ASF correction system under the current conditions.

From the information in Table 3—under the same circumstances of the weather, the wind speed and the temperature—the wind direction affected the ASF values and its influence was that the ASF values were bigger and the ranges that needed correction were bigger when the wind direction headed northeast. According to the average value of the error correction in combination with the statistical probability of the error correction interval, the selected interval range makes the statistical probability as large as possible and the average value of the error correction was calculated in this interval range. The following results can be discussed:

(1) The ASF correction system provided an average correction of 166.9449 m for the original data with wind directions towards the east-southeast.
(2) The ASF correction system provided an average correction of 218.6659 m for the original data with wind directions towards the east.
(3) The ASF correction system provided an average correction of 166.6964 m for the original data with wind directions towards the north-northeast.
(4) The ASF correction system provided an average correction of 63.7418 m for the original data with wind directions towards the west.
(5) The ASF correction system provided an average correction of 54.8815 m for the original data with wind directions towards the west-northwest.
(6) The ASF correction system provided an average correction of 321.0044 m for the original data with wind directions towards the north.

Therefore, a correction of 54.8815–321.0044 m was provided by the developed ASF correction system under the current conditions.

The information in Table 4—given the same circumstances of the wind speed, the wind direction and the temperature—shows the weather’s effects on ASF values and its influence. Foggy weather had the greatest influence on ASF values, followed by cloudy, sunny and partly cloudy weather with the least influence. According to the average value of the error correction in combination with the statistical probability of the error correction interval, the selected interval range makes the statistical probability as large as possible and the average value of the error correction was calculated in this interval range. The following results can be discussed:

(1) The ASF correction system provided an average correction of 10.4741 m for the original data when the weather was partly cloudy.
(2) The ASF correction system provided an average correction of 232.9513 m for the original data when the weather was cloudy.
(3) The ASF correction system provided an average correction of 258.2011 m for the original data when the weather was foggy.
(4) The ASF correction system provided an average correction of 218.2041 m for the original data when the weather was sunny.

Therefore, a correction of 10.4741–258.201 m was provided by the developed ASF correction system under the current conditions.

The ASF error correction values would be measured in real time and sent to the land-based positioning system when the land-based positioning system is operating. 

## 5. Results and Discussion

### 5.1. The Application of the ASF Correction System

The ASF correction system developed to correct AIS land-based positioning included two aspects: first, the ASF correction model could be obtained through the long-term statistics of the distance between the transmitter and receivers changing with environmental factors. Second, the correction system measured the test distance between two points in real time and compared it with a known real distance to obtain the time delay caused by environmental factors in the region as the real-time correction data before the correction data was broadcast to users in an effective range.

1. The data collection for constructing the ASF correction model

The transmitter and receivers were placed in a known, accurate position and receivers calculated the actual transmission delay of the VHF signal by receiving the transmit signal. In addition, according to the known accurate position and the ideal speed of light, the fixed transmission delay was calculated in the ideal condition and the ASF correction value was obtained by comparing the actual and fixed transmission delay.

The database of the VHF signal transmission data and the data processing software were established to record a large number of the actual transmission delays over a long time and to record the change of environmental factors. The relationship between the ASF of VHF signal and environmental factors was obtained by analyzing the corresponding data.

2. The real-time correction of the positioning results 

**Figure 16:**
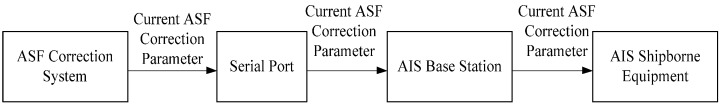
The schematic diagram of the signal connection between the ASF correction system and the AIS positioning system.

As environmental factors are very complex, the effects of the correction model were limited. The developed ASF correction system could also support the real-time correction mode. In the process of real-time correction, the receivers in the ASF correction system contrast the actual transmission delay of the VHF signal with the ideal transmission delay of the VHF signal in real time to obtain correction values. Next, the correction values were broadcast to the users by using general AIS equipment. The signal connection diagram is shown in Figure 16.

In order to obtain accurate and stable ASF correction data, it could be recommended to use the real-time correction mode of the ASF correction system, and the ASF correction model is just an alternate method when the measurement signal fails. In addition, the ASF correction data used in the practical positioning experiment mentioned in Section 5.2 could be obtained by using the real-time correction mode of the ASF correction system.

### 5.2. The Practical Positioning Experiment

**Figure 17:**
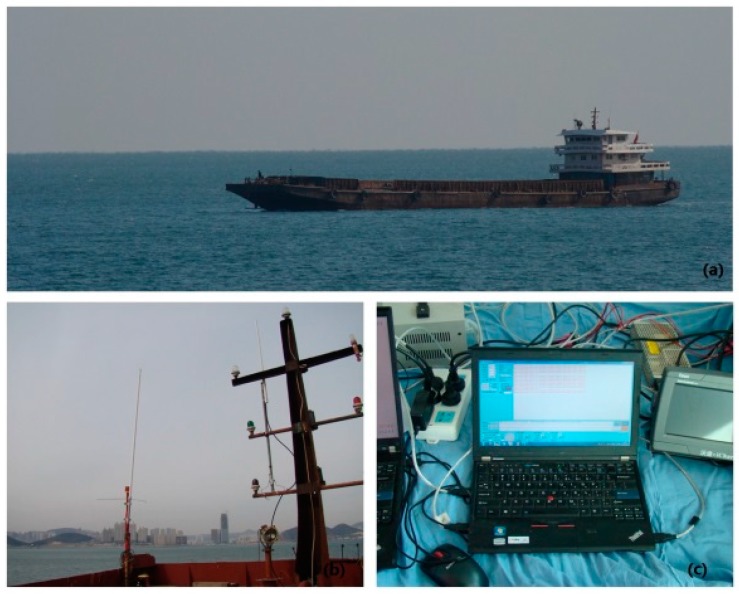
Positioning experiment scenario (**a**) The vessel for the positioning experiment; (**b**) The receiving antenna; (**c**) The received data display.

In order to verify the corrected results of the developed ASF correction system, practical experiments were conducted on the actual sea surface. The positioning experiment scenario is shown in Figure 17.

**Figure 18:**
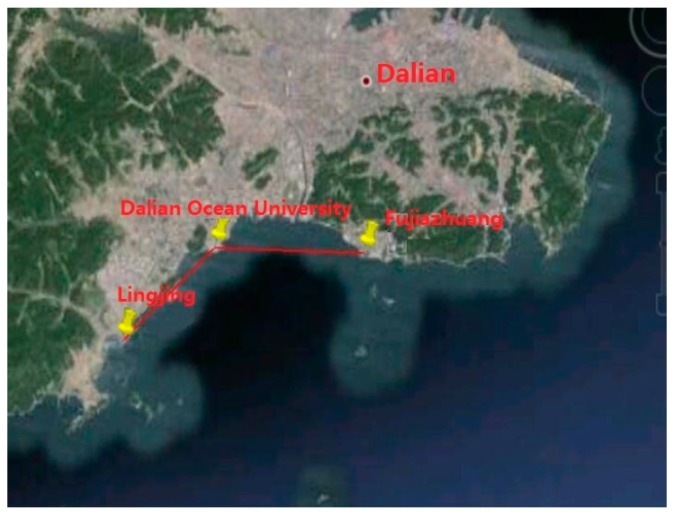
The locations of the three mark points in the static experiment.

The positioning experiments connected the ASF correction system to the self-developed AIS autonomous positioning system (AAPS), which was developed at the Navigation Institute of Dalian Maritime University in Dalian, China, where the authors work. After receiving the correction parameters, the AAPS receivers could consult the fixed measurement error of the known local receivers to correct the actual positioning data. The corrected positioning data could then be compared with the high accurate position data provided by the Dalian Maritime University–Continuously Operating Reference Station (DLMU-CORS) to obtain the corrected positioning error of the AAPS. There are three base stations in the DLMU-CORS system, located at Dalian Maritime University in Dalian, China, Mianhuang Island in Dalian, China, and Changxing Island in Dalian, China [22]. The DLMU-CORS system could provide positioning reference data in the Dalian coastal area. The positioning experiment involved a shipborne AIS receiver with the ASF correction system collecting data for 90 min, while the vessel was anchored in an area surrounded by three mark points where the GDOP was less than 1.5. The GDOP of the positioning system is investigated in [37,38,39]. The three mark points were placed at the Lingjing Hotel in Dalian, China, Dalian Ocean University in Dalian, China and the Fujiazhuang Ship Hotel in Dalian, China, respectively, as shown in Figure 18. 

**Figure 19:**
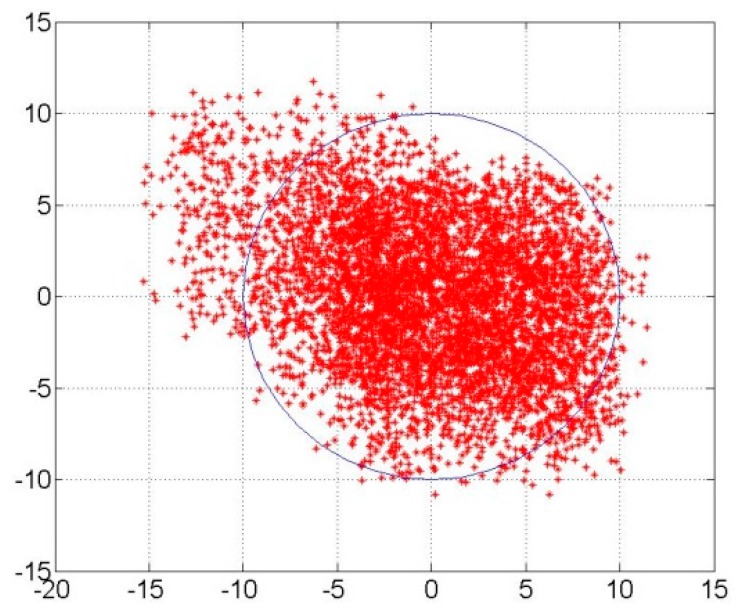
Positioning errors for the positioning experiment.

The positioning error of the positioning experiment after ASF correction is shown in Figure 19. The longitude and latitude error are represented by the horizontal and vertical axis, respectively. The mean and the root mean square (RMS) of the longitude error were −0.732 m and 3.412 m, respectively. The mean of the latitude error was 0.386 m and the RMS was 3.405 m. There were 5423 valid data collected and there were 5197 data in the blue circle. The radius of the blue circle was 10 m and only 4.2% of all the positioning results were not in this positioning error circle. Therefore, the positioning error for the position was 9.098 m (2σ) after ASF correction.

The overall design of the AAPS was presented in Reference [22] and the ASF correction technology was just described briefly. The detailed design and the development process of the ASF correction system were completed by authors of this paper. In addition, the results from this paper and Reference [22] were all about the positioning accuracy of the AAPS when using the ASF correction technology. So the details of the dynamic positioning experiment can be referred to Reference [22].

### 5.3. Discussion

In Section 4 of the paper, the range of the ASF correction was 10.4741–321.0044 m by the current test experiment and the ASF real-time correction was sent to the AAPS receiver with a GDOP of less than 1.5, and the positioning accuracy of the verification system for the AAPS was about 100 m (2σ) within the current corrected range of the ASF correction. From the experimental results of the static positioning experiment, it can be seen that the verification of the AAPS was performed to obtain high-accuracy positioning data. The positioning error after ASF correction was 9.098 m (2σ). In addition, in Reference [22], the static and dynamic positioning errors were 9.090 m (2σ) and 9.851 (2σ) using the developed ASF correction system in this paper. In conclusion, the positioning precision after ASF correction could decrease to 10 m (2σ) with a GDOP of less than 1.5.

## 6. Conclusions

This paper developed an ASF real-time correction system for the AIS signal and the system was used in processing test data from 2015. As it was restricted by the test environments, typical meteorological factors must be considered in the continuous data processing of three transceivers simultaneously obtaining high-quality test signals, which meant that the data in the paper were obtained from 25 August–3 September 2015. The developed ASF correction system measured the transmission delay of the VHF signal between the transmission system and the receiving system in different environments and statistically analyzed the relationship of the time delay and the environment to obtain the radio wave phase velocity at the VHF band, as well as other factors such as the propagation medium. From the data processing results, the following conclusions can be reached: low temperatures, fast wind speed, a northeastern wind direction and heavy fog all appeared to have great influence on the ASF value. On this basis, the statistical model of the radio wave transmission delay was established. The experiment could send real-time transmission corrections and correct the transmission delay measurements of the AIS autonomous positioning system in real time. The system test results showed that the positioning error of the ASF real-time correction system can be achieved within 10 m when the GDOP is less than 1.5. Based on the existing functions of the AIS to increase the autonomous positioning function, the AIS could meet the requirements of positioning accuracy for the land-based navigation system and could be a powerful guarantee for navigational safety which space-based and land-based backup positioning navigation systems currently perform. To promote and popularize the ASF correction system, our future work will focus on improving the ASF correction models by collecting a large amount of measured data. Once the measurement signal fails, the current correction parameter of the ASF could be obtained by the correction models which are obtained by long-term statistics.

## Figures and Tables

**Table 1:** The corresponding data change table of Figure 12.

Temperature (℃)	ASF (nano-seconds (ns))	Error Correction (meters (m))	Statistical Probability of the Error Correction Interval (%)				Error Correction Average (m)
			0–100 (m)	100–200 (m)	200–300 (m)	300–400 (m)	
22	595–954	178.43–286.07	0	4.39	95.61	0	229.0738
24	540–973	162.05–291.92	0	24.5	75.5	0	243.6178
25	622–1370	186.62–411.26	0	0.5	69.56	29.5	276.525
27	1.93–497	0.58–149.18	83.5	16.5	0	0	66.1956
28	115–661	34.51–198.31	23	77	0	0	128.1657

**Table 2:** The corresponding data change table of Figure 13.

Wind Speed (km/h)	ASF (ns)	Error Correction (m)	Statistical Probability of the Error Correction Interval (%)				Error Correction Average (m)
			0–100 (m)	100–200 (m)	200–300 (m)	300–400 (m)	
3.6	1.93–337	0.58–101.2	99.94	0.06	0	0	41.0782
7.2	21.5–400	6.44–119.93	96.78	3.22	0	0	54.8815
10.8	575–1290	172.57–385.51	0	2.5	60.06	37.44	284.662
14.4	634–1330	634–1330	0	5.61	43.5	55.94	305.8199

**Table 3:** The corresponding data change table of Figure 14.

Wind Direction	ASF (ns)	Error Correction (m)	Statistical Probability of the Error Correction Interval (%)				Error Correction Average (m)
			0–100 (m)	100–200 (m)	200–300 (m)	300–400 (m)	
East-southeast	396–727	118.76–218.21	0	96.5	3.5	0	166.9449
East	564–868	169.07–260.33	0	23.44	76.56	0	218.6659
East-northeast	384–786	115.25–235.76	0	79.22	20.87	0	166.6964
West	56.6–376	16.97–112.91	99.71	0.83	0	0	63.7418
West-northwest	21.5–400	6.44–119.9	96.78	3.22	0	0	54.8815
North	782–1260	234.58–377.32	0	0	32.33	67.67	321.0044

**Table 4:** The corresponding data change table of Figure 15.

Weather	ASF (ns)	Error Correction (m)	Statistical Probability of the Error Correction Interval (%)				Error Correction Average (m)
			0–100 (m)	100–200 (m)	200–300 (m)	300–400 (m)	
Partly cloudy	1.93–138	0.58–41.54	100	0	0	0	10.4741
Cloudy	610–930	183.11–279.05	0	3.33	96.67	0	232.9513
Foggy	673–1050	201.83–316.49	0	0	96.06	3.94	258.2011
sunny	583–872	174.92–261.5	0	12.39	87.61	0	218.2041
